# Self-Sensing with Hollow Cylindrical Transducers for Histotripsy-Enhanced Aspiration Mechanical Thrombectomy Applications

**DOI:** 10.3390/s25175417

**Published:** 2025-09-02

**Authors:** Li Gong, Alex R. Wright, Kullervo Hynynen, David E. Goertz

**Affiliations:** 1 Department of Medical Biophysics, University of Toronto, Toronto, ON M5G 1L7, Canada; khynynen@sri.utoronto.ca; 2 Physical Sciences Platform, Sunnybrook Research Institute, Toronto, ON M4N 3M5, Canada; alex.wright@sri.utoronto.ca; 3 Institute of Biomedical Engineering, University of Toronto, Toronto, ON M5S 3G9, Canada

**Keywords:** histotripsy, sonothrombolysis, cavitation, aspiration thrombectomy, ultrasound, transducer

## Abstract

**Highlights:**

**What are the main findings?**

- Miniature hollow cylindrical histotripsy transducers can detect intra-lumen cavitation without requiring external sensors.
- Voltage signals acquired across the transducer during and immediately after transmission provide insight into both the time and frequency domains.

**What is the implication of the main finding?**

- These self-sensing transducers offer a built-in method for cavitation monitoring, enabling feedback in therapeutic ultrasound applications such as histotripsy-mediated thrombectomy.

**Abstract:**

Intravascular aspiration thrombectomy catheters are widely used to treat stroke, pulmonary embolism, and deep venous thrombosis. However, their performance is frequently compromised by clot material becoming lodged within the catheter tip. To address this, we develop a novel ultrasound-enhanced aspiration catheter approach that generates cavitation within the tip to mechanically degrade clots, with a view to facilitate extraction. The design employs hollow cylindrical transducers that produce inwardly propagating cylindrical waves to generate sufficiently high pressures to perform histotripsy. This study investigates the feasibility of self-sensing cavitation detection by analyzing voltage signals across the transducer during treatment. Experiments were conducted for two transmit pulse lengths at varying driving voltages with water or clot in the lumen. Cavitation clouds within the lumen were assessed using 40 MHz ultrasound imaging. Changes in the signal envelope during the pulse body and ringdown phases occurred above the cavitation threshold, the latter being associated with more rapid wave damping in the presence of bubble clouds within the lumen. In the frequency domain, voltage-dependent cavitation signals—subharmonics, ultra-harmonics, and broadband—emerged alongside transmit pulses. This work demonstrates a highly sensitive, sensor-free method for detecting cavitation within the lumen, enabling feedback control to further improve histotripsy-assisted aspiration.

## 1. Introduction

Thrombotic large-vessel occlusions are a significant contributor to global mortality and morbidity, particularly in cases of myocardial infarction [1,2,3], ischemic stroke [4], pulmonary embolism (PE) [5], and deep venous thrombosis (DVT) [6]. Catheter-based mechanical thrombectomy techniques have become increasingly common in standard clinical practice to remove acute thrombotic occlusions in large vessels. Mechanical thrombectomy procedures are employed in the treatment of stroke [7,8,9,10,11], pulmonary embolism [5,12,13,14], and peripheral vessels [6,15,16]. One of the most widely used mechanical thrombectomy methods is aspiration, which operates by using suction to extract clot material through the lumen of a hollow catheter. In the most common form of this procedure—direct contact aspiration (DA)—the catheter tip is navigated be in contact with the proximal end of the clot and an external vacuum is applied. While this approach has significantly impacted the treatment of patients, in many circumstances it is unsuccessful or performs sub-optimally [17,18] due to thrombus clogging within the catheter or ‘corking’ at the catheter tip [19,20]. Therefore, there is a recognized need to improve the performance of aspiration mechanical thrombectomy devices, particularly their ability to ingest more challenging clots (e.g., stiffer or larger volume clots). We are developing a novel ultrasound-enhanced aspiration approach that involves the placement of a hollow cylindrical transducer (HCT) at the catheter tip. The premise is that established clinical DA procedures will be followed, but, upon the application of suction, histotripsy will be performed on clot material as it enters the HCT lumen to degrade its mechanical integrity and thereby facilitate deformation and ingestion further into the catheter. Histotripsy is an established ultrasound method that employs cavitation clouds to fractionate targeted regions of tissue into an acellular liquid [21,22,23,24]. The approach involves using very-high-pressure ultrasound to generate violent bubble clouds within the HCT focal region. There are several general classes of histotripsy, determined by the pulse lengths employed: extrinsic histotripsy typically uses ~1–2 µs pulses and requires the highest pressure levels; shock scattering histotripsy uses 5–20 µs pulses; and ‘boiling’ histotripsy involves 1–100 ms pulses and requires the lowest pressure level due to pulse-scale thermal elevations. With few exceptions [25,26,27], large aperture extracorporeal spherically focused transducer configurations are used to achieve the pressure levels required to create cavitation clouds.

With the proposed aspiration technique, sufficiently high pressures must be achieved within the lumen of HCTs that are of a scale compatible with intravascular catheters. In our prior work [28], we demonstrated that cavitation clouds could be generated within a radially polarized HCT (3.3/2.5 mm outer/inner diameter) when operating within its ‘thickness’ mode resonant frequency bandwidth. The associated transducer vibrations produce predominantly cylindrical waves and a high-pressure zone along the HCT lumen axis of symmetry. In particular, when operating at a standing wave frequency, constructive interference can increase the effective focal gain, resulting in increased internal pressures (estimated > 20 MPa). In this work, cavitation within the lumen was confirmed with a hydrophone situated external to the HCT lumen and high-frequency (40 MHz) ultrasound imaging. The pulse lengths employed (10 and 100 µs) were sufficiently long to permit the development of standing wave. This was supported by hydrophone data that revealed pressure buildup at the outset of electrical pulse stimulation that was associated with the development of the interference pattern, and a decay (ringdown) following the end of the pulse associated with the dissipation of internal reflections. In a more recent work [29], we demonstrated the feasibility of performing histotripsy within clots situated in an HCT lumen.

An important element of any cavitation-mediated therapeutic ultrasound approach is monitoring and controlling the level and characteristics of cavitation. Cavitation monitoring and control methods have been widely investigated in the setting of microbubble-mediated drug delivery [30,31,32,33]. Generally, these involve using receive transducers, separate from the transmit transducers, to passively detect and, in some cases, spatially map cavitation emissions arising from the therapy pulses. In histotripsy, the primary approach to cavitation monitoring to date has involved situating an ultrasound imaging transducer within the therapy transducer aperture. B-scan ultrasound imaging is then used to ‘actively’ (pulse-echo) detect bubble clouds that form during the treatment process. The imaging information is used for targeting purposes, confirming the formation and extent of lesions, and potentially adjusting the transmit conditions to ensure sufficient cavitation activity. More recently, efforts are underway to develop transmit–receiver arrays compatible with short-pulse (extrinsic) histotripsy. In [34,35], a subset of elements was configured with both transmit and receive capabilities, enabled by the short duration of the transmitted pulses relative to the propagation time to regions of interest, resulting in time separation between the transmit and receive signals.

For the proposed ultrasound-enhanced aspiration approach, as with conventional histotripsy, the ability to detect and monitor induced cavitation will be necessary to ensure that effective treatments are occurring and to potentially adjust transmit parameters as necessary. As the intended application areas are in the setting of stroke, PE, and the peripheral vasculature, using a separate B-scan imaging transducer to monitor cavitation within the HCT lumen is not viable. Furthermore, due to the spatial lengths of the pulses employed relative to the lumen diameter, there will not be temporal separation between the transmit and receive signals [28]. In the present study, we will investigate the use of voltage signals measured across the transmit transducer to monitor cavitation induced within the lumen of the HCT. In the setting of boiling histotripsy conditions, using a single-element large aperture spherically focused transducer, there have been several reports of fluctuations in the voltage or power across the transducer after the onset of cavitation within the focal region [36,37]. These fluctuations result from the signals across the transducer being a superposition of the applied transmit voltage and the arrival of emissions and reflections/scattering from the cavitation cloud at the focal region. Outside the biomedical ultrasound field, particularly in the area of sonochemistry, there have been numerous reports of examining measured transducer signals (voltage and current) to monitor cavitation with long-duration pulses (>100 ms) [38,39,40,41]. This work has been carried out for acoustic horn or larger-scale cylindrical focusing configurations. It has been shown that the measured signals can detect the presence of inertial cavitation, harmonics, and subharmonics [42,43]. This is often referred to as ‘self-sensing’ due to the dual use of the transducer as a transmitter and receiver, with the target cavitation signals being concurrent with the transmission stimulation signal.

Here, we investigate the use of self-sensing approaches to detect and monitor cavitation within an intravascular scale HCT. Following the conditions employed in recent work (between 10 and 100 µs pulses) [28], signals measured across the HCT will be assessed over a range of applied voltages from sub-cavitation to cavitation levels. The temporal (pulse-body and ringdown) and spectral aspects of the pulses will be assessed. The presence of cavitation will be confirmed using high-frequency ultrasound imaging. The majority of experiments will be conducted with water-filled lumens, followed by a proof-of-principle experiment with thrombus-filled lumens.

## 2. Materials and Methods

### 2.1. Transducer Configuration

The transducer used for experiments was a radially polarized HCT with an outer diameter of 3.3 mm and an inner diameter of 2.5 mm, with a length of 2.5 mm, as employed in [28]. The transducer material was DL-47 (DeL Piezo Specialties, West Palm Beach, FL, USA), a hard PZT ceramic suitable for high-power applications due to its high mechanical quality factor, relatively high dielectric constant, and low dielectric loss.

**Figure 1:**
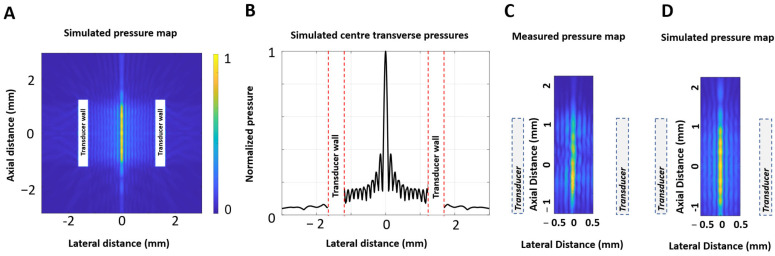
Simulated sagittal 2D pressure map (**A**) and pressure across the HCT transverse axis of symmetry (**B**) operating at 6.17 MHz in water. Detailed views of measured (**C**) and simulated (**D**) pressures within the HCT lumen show qualitatively consistent profiles.

The transducer had a micro-coaxial wire attached to the inner signal and outer ground electrodes using silver epoxy (Epotek H20E, Epoxy Technology Inc., Billerica, MA, USA) and a catheter liner (PTFE Sub-Lite-Wall™ Liner 0.095” ID 0.0015” wall thickness, Zeus Company Inc., San Jose, SC, USA) attached to its inner wall using epoxy (Epotek 301, Epoxy Technology Inc., Billerica, MA, USA). This design is capable of achieving high internal pressures and generating cavitation clouds within the HCT lumen, as reported in previous work [28]. Finite element simulations of the HCT, including the liner and epoxy layers, were performed in OnScale^TM^, as per the methods previously described in [28] (material properties in Supplementary Table S1). Figure 1A,B depict the simulated normalized pressure map and lateral pressure distribution within the lumen for the transducer using 10 µs pulses at 6.17 MHz in water. These results show a narrow high-pressure region along the central axis of the cylinder and the presence of side lobes that decay in amplitude towards the HCT lumen walls. Figure 1C shows fiber-optic hydrophone (10 μm active region, Precision Acoustics Inc., Dorset, UK)-based measurements of the internal pressure field, and Figure 1D highlights a matching detailed view of the simulation for comparison, emphasizing the similarities between the two results. Absolute pressures are not measured here due to the directional sensitivity of the hydrophone, coupled with the angle of incidence of the wavefronts being perpendicular to the hydrophone axis, as discussed in [28]. It was previously established that sufficiently high pressure levels can be achieved to initiate cavitation within the lumen, and the primary purpose of the present study is to examine self-sensing-based methods to detect this form of cavitation. Supplementary Figure S1 shows a comparison of simulated profiles for 10 µs and 100 µs pulses. They exhibit similar spatial distributions to peak pressures, which is expected, as the luminal standing-wave pattern is fully developed for the particular HCT employed with pulse lengths of 10 µs or greater [28].

### 2.2. Cavitation Observation with High-Speed Ultrasound Imaging

#### 2.2.1. Experimental Configuration

**Figure 2:**
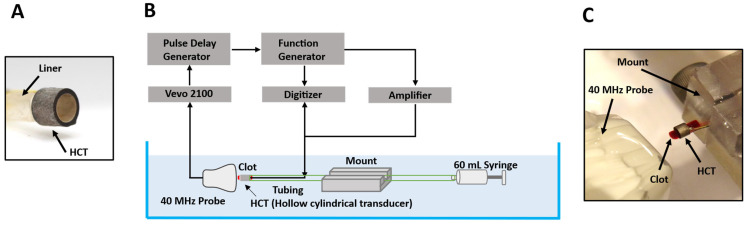
(**A**) Image of the constructed transducer with attached liner. (**B**) Schematic overview of experimental configuration. (**C**) Photo of experimental setup with a clot present in the HCT lumen.

The experimental setup is shown in Figure 2, where an HCT and imaging transducer were situated in a tank of gas-equilibrated deionized water held at 30 °C. A 2.5mm diameter PTFE liner (Sub-Lite-Wall™ Liner 0.095” ID 0.0015” wall thickness, Zeus Company Inc., Orangeburg, SC, USA) was epoxied to the lumen surface of HCT to mimic the eventual intended catheter configuration, as well as facilitate positioning within the tank. A Vevo 2100 imaging system with a 40 MHz probe (MS550D, FUJIFILM VisualSonics Inc., Toronto, ON, Canada) was positioned to image the central longitudinal cross-section of the HCT lumen. The framerate was set to 916 Hz, which was the system’s maximum for a field of view that captured the entirety of the lumen diameter across the full transducer length. The imaging system was synchronized with treatment pulses using a pulse delay generator (Model 575, Berkeley Nucleonics Corp., San Rafael, CA, USA) to ensure that every treatment pulse occurred at the same time point during each frame. The transducer was driven by a function generator (AFG3102, Tektronix, Beaverton, OR, USA) and an RF amplifier (A150, E&I, Jericho, NY, USA). The voltage across the HCT was measured using a 100:1 voltage probe and a digitizer PicoScope 5242B (Pico Technology, England, UK) at a sample rate of 125 MS/s (14 bits). The applied voltage amplitudes reported are the average steady-state peak voltages (V_p_) measured across the transducer.

#### 2.2.2. Transmit Frequency Selections

The impedance of HCT in water was measured from 300 Hz to 8 MHz in 5 kHz steps using a network analyzer (AA-30.ZERO, RigExpert, Kyiv, Ukraine) with both water and a clot in the HCT lumen. Within the thickness mode bandwidth of the HCT geometry, there are a number of prominent minima. These are associated with standing wave frequencies within the lumen, and were previously shown to correspond to local (frequency) pressure maxima [28]. As in [28], the transmit frequency was selected by observing the HCT lumen under B-mode imaging while sending ‘treatment’ pulses (82 V_p_ voltage, 10 µs pulses) at each frequency, corresponding to a minimum within the thickness mode bandwidth. The minima frequency that resulted in the largest cavitation cloud on B-scan imaging was then employed as the transmit frequency for the experiments. This process was carried out for both water-filled and clot-filled lumens to determine the most appropriate operating frequency for each case as the impedance minima differed, as described below.

#### 2.2.3. Experimental Protocol

For experiments with water in the lumen, the transducer was operated at 6.17 MHz, and pulse lengths of 10 µs and 100 µs were tested. A total of 100 pulses were sent per sequence, with a pulse interval of 10.92 ms, such that one pulse was imaged every 10 frames for 1000 frames. A total of 26 applied voltages ranging from 19 to 82 V_p_ were tested. Each 100-pulse sequence was repeated three times at each voltage and pulse length, resulting in 300 pulses per voltage condition. We note that the maximum voltage applied here corresponds closely to that reported in [28] (82 V_p_), which resulted in high levels of cavitation and did not result in any change in the performance of the transducer over time. The lowest tested voltage (19 V_p_) did not produce cavitation in any configuration, again consistent with previous work [28].

For experiments with a clot in the lumen, a frequency of 6.43 MHz was used. A pulse length of 10 µs and a pulse interval of 1.09 ms were used for 916 pulses, resulting in a pulse every frame for 916 frames. A total of 5 clots were tested in this manner. For each clot, the treatment was performed 5 times at 19 V_p_ (sub-cavitation threshold) and then once at 82 V_p_. After treatment, the clots were extracted and bisected for optical imaging under a microscope.

### 2.3. Cavitation Threshold

B-mode imaging data was used to determine whether cavitation occurred on a given pulse. Each frame, corresponding to a specific treatment pulse, was manually assessed for the presence or absence of visible cavitation, which manifests as hyperechoic regions within the transducer lumen. To determine cavitation probability in water, for a given applied voltage, the ratio of the number of pulses which had visible cavitation activity to the number of total pulses was calculated. This probability, as a function of applied voltage, was fitted to a sigmoid curve. The voltage value at a probability of 0.5 was defined as the cavitation threshold voltage.

### 2.4. Self-Sensing Signals Analysis

Signal analysis was performed using MATLAB R2021a (MathWorks, Natick, MA, USA) and GraphPad Prism 10 (GraphPad Software, San Diego, CA, USA). For each pulse, time domain voltage signals acquired from the transducer can be divided into three segments: pre-pulse (noise only), ‘pulse body’ (during transmit), and ‘ringdown’ (post-transmit). In this study, ringdown refers to the period of decay following the end of the transmit pulse (applied voltage), which is primarily associated with the dissipation of internal reflections within the HCT lumen. Note that such reflections are distinct from transducer ringdown, which arises from residual mechanical vibrations in the transducer material itself.

#### 2.4.1. Time Domain Analysis

For time domain signal analysis, two temporal windows were examined: a pulse body window and a ringdown window. The pulse body window (duration 1.2 μs for water and 1.67 μs for clot) began 3 cycles after the start of the transmit pulse (applied voltage) to allow for a steady state to be reached. The ringdown window was 2 μs in duration, beginning after 3 cycles following the cessation of the transmit pulse. Note that there was a range of window durations that was evaluated and produced relatively similar results. For each of these windows, the envelope of the signal was squared and then summed over the window duration to obtain an estimate of power. The ratio of these resulting metrics (ringdown to pulse body) was then calculated to provide a measure of the ringdown signal level relative to the pulse body amplitude, which we define here as the *ringdown ratio*. Employing this ratio effectively normalizes the results to the different driving voltages tested.

For the water-in-lumen case, to provide an indication of the impact of cavitation on the pulse-to-pulse variability in the ringdown ratio, the variance of this metric was calculated over the 300-pulse sets at each applied voltage.

#### 2.4.2. Spectral Analysis

Frequency domain analysis was conducted on the pulse body portion, during which active cavitation could occur. The 10 µs Blackman windows were used for 10 µs and 100 µs Tukey windows with a cosine fraction of 0.25 were used for 100 µs pulses. For 10 µs pulses, zero padding was employed in order to match the frequency resolution of the 100 µs windows. Fast Fourier transforms were performed on each windowed signal, and the spectra were power averaged for each applied voltage. The same process was applied to segments of signal acquired pre-trigger prior to the arrival of the transmit pulse in order to generate baseline noise-only spectra.

As a means of quantifying cavitation, the sum of the power was calculated over the frequency windows surrounding the fundamental frequency (i.e., 6.03–6.34 MHz for 10 µs pulses and 6.15–6.20 MHz for 100 µs pulses in water), ultra-harmonic frequency (i.e., 9.21–9.31 MHz for both pulse lengths in water and 9.55–9.67 MHz with a clot), and a frequency range corresponding to broadband signals (i.e., 19.30–19.70 MHz for 10 µs pulses in water; 5.13–5.40 and 7.00–7.30 MHz for 100 µs pulses in water; 5.45–5.60 and 7.45–7.70 MHz with a clot).

### 2.5. Blood Clot Preparation

Clots were prepared in a manner similar to previous sonothrombolysis studies [29,43,44,45,46,47,48]. Clots were prepared from blood extracted from the femoral vein of a pig and were captured in 3.2% sodium citrate vacutainers (BD Vacutainer™, Thermo Fisher Scientific Inc., Waltham, MA, USA). Clots were formed in 2 mL borosilicate pipettes (Fisherbrand™ Disposable Borosilicate Glass Pasteur Pipets, Thermo Fisher Scientific Inc., Waltham, MA, USA) after mixing anticoagulated blood with 100 mMol/L CaCl_2_ at a ratio of 1 mL of anticoagulated blood to 200 µL of CaCl_2_. The sealed pipettes were incubated in a water bath at 37 °C for 3 h and then transferred to a fridge at 4 °C for 3 days. The resulting retracted clots were extracted and cut into homogenous 5 mm lengths prior to the experiments.

## 3. Results

### 3.1. Impedance and Frequency Selection

**Figure 3:**
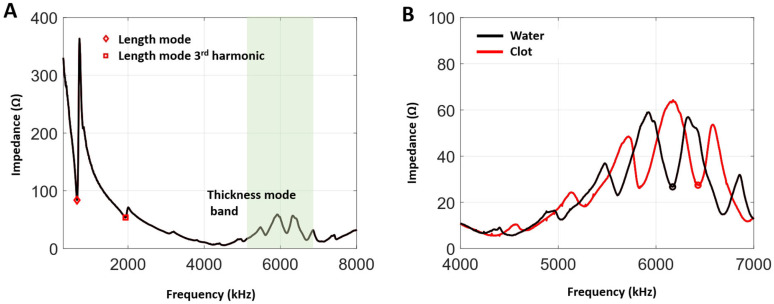
The measured electrical impedances as a function of frequency in a range of 300–8000 kHz (**A**) and 4000–7000 kHz (**B**), with either water or clot occupying the transducer lumen. Minima at 660 kHz and 1.93 MHz correspond to the length mode and its third harmonic. The transmit frequencies used for clot treatments and treatments with water in the lumen are highlighted with circle markers in (**B**).

The impedance as a function of frequency for the water-immersed HCT is shown in Figure 3A. Local minima associated with the fundamental length mode and its third harmonic appear at 660 kHz and 1.93 MHz, along with a more complex series of peaks and troughs near the thickness mode bandwidth (4.9–7 MHz). Figure 3B shows measurements for both water- and clot-filled lumens within the thickness mode bandwidth. The minima are each associated with a standing-wave frequency [28]. Having a clot in the lumen shifts the frequencies of these minima higher relative to the water case.

Following the previously described methods for transmit frequency selection, the thickness mode minima identified above were evaluated for their capacity to generate cavitation. The frequencies selected for the water-in-lumen and clot-in-lumen cases were 6.17 and 6.43 MHz, respectively. The difference between these two transmit frequencies is consistent with the minima shift in the impedance curve.

### 3.2. Cavitation Imaging and Cavitation Probability with Water-Filled Lumen

**Figure 4:**
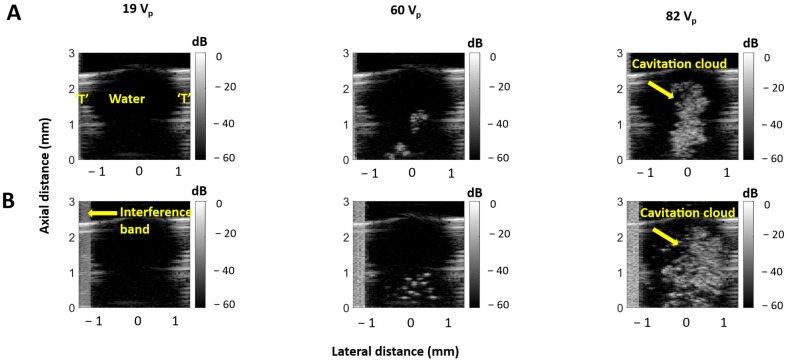
B-mode imaging frames of the lumen of an HCT in water with 10 µs (**A**) and 100 µs pulses (**B**). ‘T’ indicates HCT wall.

**Figure 5:**
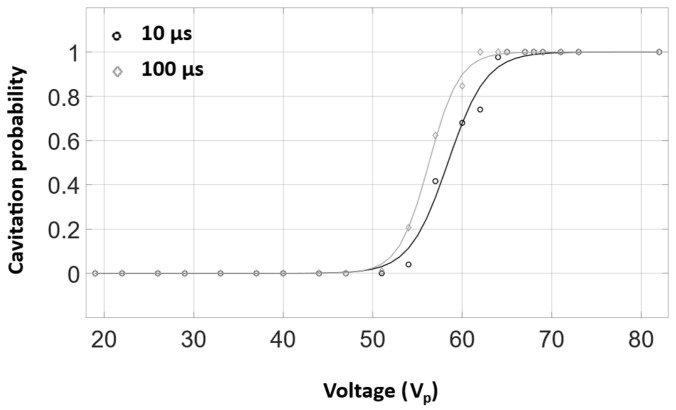
Cavitation probability in gas-equilibrated water as a function of applied voltage, as measured using B-mode imaging for both 10 and 100 µs pulses.

Figure 4 displays example B-mode imaging frames of the HCT operating in water. At low voltages, the lumen is hypo-echogenic, consistent with the absence of cavitation [28]. As the voltage increases, cavitation clouds begin to appear, and, at high voltages, there is strong cavitation activity, although it remains confined within the transducer lumen. This trend holds for both the 10 and 100 µs pulses, although the 100 µs pulses have larger cavitation clouds at higher voltages. The cavitation probability in water is shown in Figure 5. No cavitation was observed below 54 V_p_ for either the 10 or 100 µs pulses. For both pulse lengths, above 65 V_p_ cavitation was observed for every pulse.

### 3.3. Self-Sensing Signals in Water-Filled Lumens

**Figure 6:**
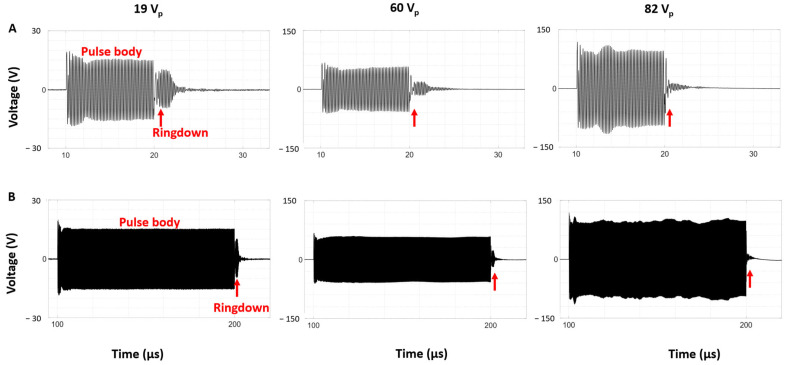
Voltages measured across the transducer during (**A**) 10 µs and (**B**) 100 µs pulses at different applied voltages in water. The red arrows indicate the ringdown portion of the signals.

Figure 6 presents example time domain voltage signals for both 10 and 100 µs pulses and at 19, 60, and 82 V_p_ in water. An amount of 19 V was shown to be well below the cavitation threshold (Figure 5), and no cavitation is observed with B-mode imaging (Figure 4), while 60 V_p_ is around the cavitation threshold voltage range and 82 V_p_ is substantially above the cavitation threshold. Comparing the envelopes of the voltages at each applied voltage reveals differences in the ratios of the amplitude of the envelopes of the ringdown portions to the pulse bodies. In particular, the relative ringdown ratios are progressively lower at the higher voltages—where cavitation is present—relative to the 19 V_p_ case (sub-cavitation threshold). In addition, it can be seen that fluctuations are present in the envelope of the pulse body in the presence of cavitation. This is consistent for both the 10 and 100 µs pulses.

**Figure 7:**
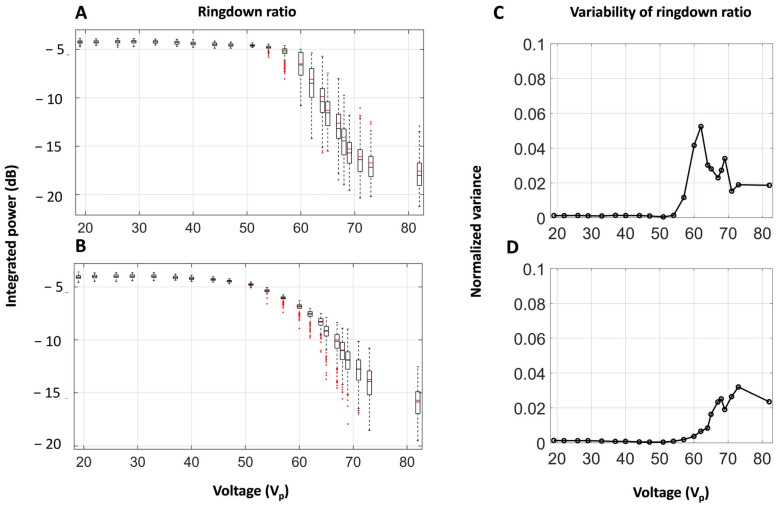
Box plots of the ringdown ratio as a function of voltage for (**A**) 10 µs and (**B**) 100 µs pulses in water. The results are normalized to the mean ringdown ratios at 19 V for each dataset, and the box plots identify the median as the central marks, the 25th and 75th percentiles as the box edges, the most extreme non-outlier datapoints as the whiskers, and individual outliers as red markers. (**C**) and (**D**) show the variance of these ringdown ratios as a function of voltage for the 10 µs and 100 µs pulses, respectively.

Figure 7A,B shows a box plot of the ringdown ratio as a function of applied voltage. For both 10 and 100 µs pulses, at lower voltages—below the cavitation threshold—the ratio remains constant. As the cavitation threshold voltage is approached, the ratio begins to decrease with increasing applied voltage. Figure 7C,D shows the variance in the ringdown ratio as a function of voltage. The variance increases at the onset of cavitation before plateauing at approximately 64 V_p_ in the 10 µs case and 67 V_p_ in the 100 µs case.

**Figure 8:**
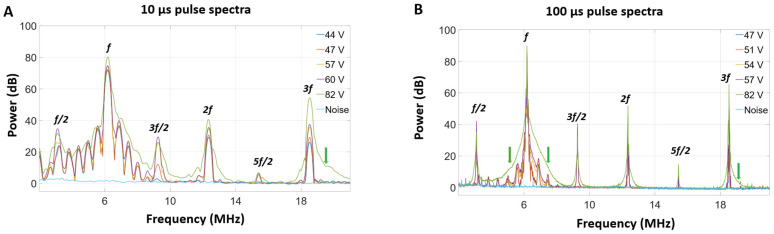
The spectra of (**A**) 10 µs and (**B**) 100 µs pulses at different voltages in water. The green arrows point to regions of broadband emissions which are visible at 82 V_p_; f indicates the fundamental frequency of transmit pulses (6.17 MHz).

Pulse body spectra at select voltages are shown in Figure 8. Sub-harmonic, ultra-harmonic, and broadband signals emerge for voltages around and above the cavitation threshold voltage for both the 10 and 100 µs pulses.

**Figure 9:**
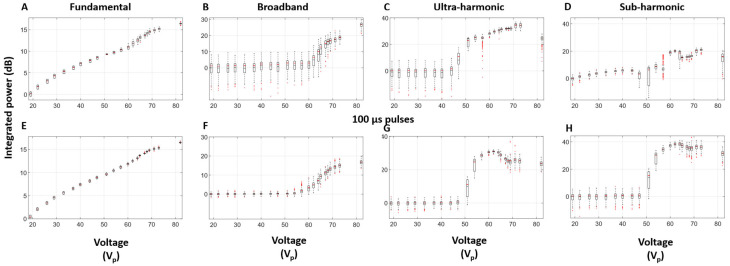
Box plots of integrated power of specific frequency bands of the transducer voltage spectra as a function of applied voltage in water. The broadband (**B**,**F**), ultra-harmonic (**C**,**G**), and sub-harmonic (**D**,**H**) for both 10 µs and 100 µs pulses show a threshold behavior, while the fundamental (**A**,**E**) show a monotonically increasing behavior. The integrated powers are normalized to the mean values at 19 V_p_ within each dataset.

The quantification of selected frequency bands as a function of voltage is shown in Figure 9. For 10 µs pulses, the fundamental frequency signal increases monotonically as a function of applied voltage. An increase in broadband signal becomes apparent from 62 V_p_ and continues to rise with applied voltage and begins to plateau around 69 V_p_. The ultra-harmonic signal exhibits a similar pattern, but with a lower voltage onset (47 V_p_). The sub-harmonic signal follows a more complex pattern, but also suggests the presence of an onset voltage and eventual plateau. This complexity may be due to overlap with contributions from thickness mode frequencies. The overall trends for the 100 µs pulses share many of these characteristics. Notable differences include lower standard deviations below the cavitation threshold, which results in a more distinct cavitation threshold curve. Interestingly, both the ultra- and sub-harmonic signals have more similar curve features and also undergo a reduction at the highest voltage levels.

### 3.4. Self-Sensing Signals During Clot Treatments

**Figure 10:**
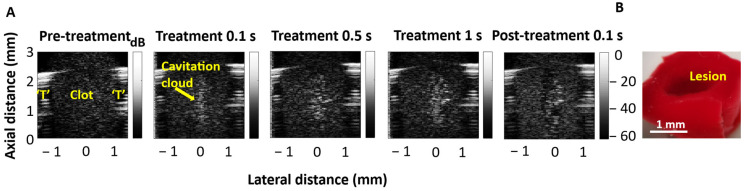
(**A**) Representative B-mode imaging of a cross-section of a clot situated in the transducer before, during, and after treatment. ‘T’ represents HCT walls. Treatment parameters were 10 µs pulse length, 1000 Hz PRF, and 1 s treatment duration. Before treatment, the clot is visible as uniform speckle bounded by the side walls of the transducer. During treatment, the cavitation cloud is visible as a hyperechoic region along the central axis of the HCT. (**B**) Immediately post-treatment, the hypoechoic region is visible over the same area of the cavitation cloud, indicating the generation of a lesion, which is confirmed by the bisected clot.

Figure 10 shows representative B-mode images of the treatment (82 V_p_) of a clot in the lumen of the HCT. A central cavitation cloud is visible from the beginning of the treatment, and, post-treatment, a lesion in the same location is visible. The bisected extracted clot shows the lesion hole under optical imaging.

**Figure 11:**
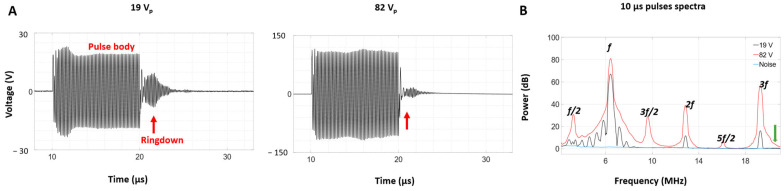
Voltage signals across the HCT with a clot in the lumen at (**A**) 19 V_p_ and (**B**) 82 V_p_, and (**C**) the spectra of these signals. The red arrows on the time domain traces indicate the ringdown portions of the signals, and the green arrows on the spectra indicate broadband emissions at 82 V_p_.

Figure 11A shows representative time domain voltage signals during the exposure of a clot (1 s duration) at applied voltages of 19 (sub-cavitation) or 82 V_p_, along with their associated frequency domain spectra. Similarly to the case for the water-filled HCT lumen, the ringdown ratio decreased significantly from 19 to 82 V_p_. The spectra shown in Figure 11B also indicate that sub-harmonic and ultra-harmonic signals are present at 82 V_p_ which are not detected at 19 V_p_.

**Figure 12:**
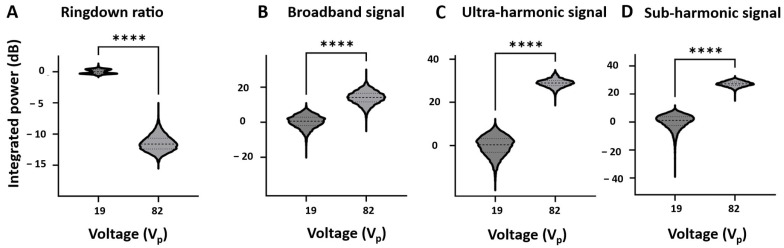
Violin plots comparing the integrated power for (**A**) the ringdown ratio in the time domain, and the broadband, ultra-harmonic, and sub-harmonic (**B**–**D**) of specific frequency bands of the HCT voltage signal spectra at both 19 and 82 V_p_ with a clot in the lumen. The integrated power is normalized to the mean value at 19 V_p_. Dashed lines represent the median and quantiles. **** indicates statistical significance between groups with *p* < 0.0001 using a two-tailed unpaired *t*-test.

The quantification of these features across the five clots tested is presented in Figure 12 as violin plots. Metrics using ringdown ratio, broadband signals, sub-harmonic, and ultra-harmonic signals all demonstrate significant (*p* < 0.0001) differences in integrated power between 19 and 82 V_p_.

## 4. Discussion

In conventional extracorporeal histotripsy systems, the detection and monitoring of cavitation is an important element of the treatment process. Active and passive cavitation detection approaches continue to be investigated to facilitate this process. In the setting of the ultrasound (histotripsy)-enhanced aspiration approach that we are developing, cavitation detection also has potential to provide valuable information for treatment monitoring and control. The approach taken here is to examine the use of the transmit transducer for this purpose, which, for a disposable catheter, would have notable advantages, including simplicity and cost-effectiveness. The results collectively demonstrate the use of measured voltage signals across an HCT to detect cavitation induced within its lumen. The measured signals underwent both temporal and spectral analyses, which were conducted by separating the signals into pulse body and ringdown segments. The cavitation-related experiments were supplemented by electrical impedance measurements across the HCT.

For water-filled lumens, fluctuations in the envelope of the pulse body became evident following the onset of cavitation, which is consistent with previous reports for spherically focused transducers in the setting of boiling histotripsy conditions [37,49]. These fluctuations arise from the superposition of signals associated with cavitation (emissions, reflections/scattering) and the applied transmit signal voltage.

To extend these observations beyond time domain fluctuations, pulse body spectral analysis was conducted. At lower voltages—below the imaging-detected cavitation threshold—the spectra feature a prominent peak at the transmit frequency alongside a number of smaller adjacent peaks/lobes within the thickness mode bandwidth. The side lobes may be associated with standing wave frequencies, as well as Fourier analysis artifacts. Multiple harmonics (second, third) are also present, which can be associated with nonlinear bubble behavior, as well as contributions from nonlinear propagation. The onset of detected sub-harmonic and ultra-harmonic signals (1/2, 3/2, and 5/2) at higher voltages is consistent with pressure threshold-dependent bubble behavior. The emergence of broadband signals followed a similar pattern, albeit with a higher voltage onset and variance. In interpreting the broadband signal, it should be noted that the frequency ranges selected for quantification were within the thickness mode range (between the local peaks) and adjacent to the third harmonic signal peak, as shown in Supplementary Figure S2. In [28], hydrophone measurements performed adjacent to the lumen at higher voltages demonstrated a broadband appearance across a wide range of frequencies. This was not observed in the self-sensing HCT spectra, which is attributed to the frequency response of the transducer when exposed to emissions impinging on its lumen surface. As the frequency ranges employed were in proximity to the fundamental and third harmonic peaks, there may be contributions from spectral broadening of these peaks in addition to general broadband noise that can be associated with inertial cavitation. This effect may have been more prominent for the 10 µs relative to the 100 µs case due to the impact of Fourier transform-related spectral broadening. Overall, the results shown in Figure 9 indicate that the selected sub-harmonic, ultra-harmonic, and broadband signals are substantially elevated over baseline values in the presence of cavitation. This is further supported by the analysis of Supplementary Figure S3, which shows that these signals are significantly elevated at high voltages relative to lower sub-cavitation voltages (e.g., 82 V_p_ vs. 19 V_p_).

It was also observed that the ringdown ratio remained relatively flat at lower voltages (~<50 V_p_), but, after the onset of detected cavitation, it began to drop precipitously. In [28], the simulated and hydrophone-measured pressure waveforms external to the transducer exhibited a stepped decay over time following the cessation of the stimulating pulse, which was attributed to the dissipation of internally reflected cylindrical waves. The measured ringdown signals are therefore interpreted to be predominantly associated with internally reflecting waves that dissipate over time. The progressive reduction in amplitude of the ringdown ratio with increasing levels of cavitation is hypothesized to result from interactions with the internally reflected waves with the bubble clouds. That is, it can be expected that waves encountering clouds will be scattered and reflected and lose energy through absorption processes. Unlike self-sensing based cavitation detection in the context of spherically focused transducers, the reduced ringdown ratio is a feature that is distinct to the HCT configuration, where internally reflected waves can be generated. It is also notable that cavitation detection via the ringdown analysis presented here has been previously reported with large-scale HCTs (>10 cm typically), such as those employed in sonochemistry [38,39,40,41]. Indeed, in sonochemistry configurations, continuous waves or very long pulses are employed and the emphasis is to assess cavitation signatures in the frequency domain. In the application being investigated here, which employs shorter pulses, ringdown analysis appears to robustly detect the presence of cavitation within the lumen.

Impedance spectra measurements show a pattern of minima within the thickness mode bandwidth for both water- and clot-filled lumens. These were previously shown in [28] (for water-filled lumens) to be associated with standing-wave frequencies, which produced higher pressure levels within the lumen due to the effects of constructive interference. As in [28], the operating frequency was selected based on the frequency that produced the highest level of cavitation, based on B-mode imaging. The locations of impedance minima were found to differ between water- and clot-filled lumens (Figure 2B). This can be attributed to differences in the compressional wave propagation velocity: clots have a higher wave speed than water [50], which, in turn, shifts the frequency at which minima occur. In a clinical setting, clots can have a range of compositions and ages, which can be expected to give rise to variations in the propagation velocity. Although a dedicated network analyzer was used in this study to measure electrical impedance, an alternate approach could involve monitoring the current, in addition to the voltage, to derive the impedance.

The proof-of-principle experiments to examine self-sensing signals in the context of clot erosion were conducted at two voltages: one below the cavitation threshold and one at the maximum voltage. The high-voltage exposures resulted in the production of a lesion within the clots, as observed in our recent study [29]. The cavitation and lesions were preferentially initiated in the central portion of the lumen, where pressure levels are highest (Figure 1). This is consistent with the results reported in [28,29]. No cavitation was observed lateral to the transducer due to the low-pressure levels outside the transducer lumen. The average spectra during the course of the treatment had similar features to the water-filled lumens. A comparison of the ringdown, sub-harmonic, ultra-harmonic, and broadband signals between the two voltages showed highly significant differences. This supports the notion that these are candidate metrics to detect cavitation during clot treatments.

The results of this work suggest that both electrical impedance and voltage signals of an intravascular-scale HCT have the potential to provide information that is relevant to the monitoring and control of ultrasound-enhanced aspiration techniques that are being developed. For example, the impedance spectra could be employed to identify candidate transmit frequencies (e.g., at minima within the thickness bandwidth) when the clot has entered the lumen. The candidate frequencies could then be tested to evaluate their capacity to produce cavitation. As B-mode imaging of the lumen cannot be performed in a clinical setting, the degree of cavitation at the candidate frequencies could be assessed by self-sensing cavitation metrics, such as those evaluated in this study, to select the operating frequency. Furthermore, an examination of the evolution of these signals over time during treatments—both for ‘static’ and aspirating clots—is necessary to determine their possible role in dynamically adapting exposures during the course of treatments. Work is underway in relation to examining the impact of clot erosion on aspiration success rate of in vitro clots.

The implementation of self-sensing signal-based monitoring and control methods would necessarily have to undergo pre-clinical experiments in relevant animal models and, eventually, clinical testing. While valuable, benchtop experiments cannot recapitulate all relevant physiologic and anatomic conditions that are present in vivo. As the signals are derived predominantly from within the catheter lumen, it is not anticipated that the presence of a vessel wall or relative motion between the catheter and wall would significantly impact their characteristics. The presence of blood may alter the signals, as there are differences in the properties (e.g., attenuation and speed of sound [50]) between blood, clots, and saline. There may also be different pressure thresholds for cavitation in vivo, although this is not expected to significantly impact the methods described above. Ultimately, this work must be conducted using fully constructed catheters.

Catheter-based ultrasound systems have been developed for treating large-vessel occlusions, most notably the clinically approved EKOS catheter used for DVT [51,52] and PE [53]. The EKOS catheter must be inserted directly into the clot, where it delivers thrombolytic drugs through side ports and applies low-intensity ultrasound to improve enzymatic activity, typically over several hours. The use of microbubbles to enhance the performance of the EKOS catheter is also being investigated [54,55]. There have also been research efforts to develop forward-looking ultrasound catheters that apply energy at the proximal clot face, generally in conjunction with cavitation agents such as microbubbles and nanodroplets [55,56,57,58,59,60,61]. These approaches rely on the progressive in situ erosion of thrombus to restore flow, assuming the downstream clearance of clot fragments. In contrast, the objective of ultrasound-enhanced aspiration is to use histotripsy improve the performance of an established and widely used mechanical thrombectomy procedure that restores blood flow by removing thrombotic vascular occlusions.

## Data Availability

The original contributions presented in this study are included in the article/Supplementary Material. Further inquiries can be directed to the corresponding author(s).

## Supplementary Materials

The following supporting information can be downloaded at: https://www.mdpi.com/article/10.3390/s25175417/s1, Figure S1: Simulated pressure maps using 10 µs (left) and 100 µs (right) pulses at 6.17 MHz in water. Pressures are normalized to the peak pressure within the field. Figure S2: Box plots of integrated power of specific frequency bands of the transducer voltage spectra as a function of applied voltage in water for both 10 µs and 100 µs pulses. The 2nd harmonic (A,D) increases in a linear behavior. The 3rd harmonic (B,E) signal exhibits an inflection at a sub-cavitation voltage, while the 4th harmonic (C,F) starts to increase at voltage over the cavitation threshold. The integrated powers are normalized to the mean value at 19 V_p_ within each dataset. Figure S3: Violin plots for both 10 µs and 100 µs pulses comparing the integrated power for (A, E) the ringdown ratio in time domain and the broadband (B,F), ultra-harmonic (C,G), sub-harmonic (D,H) of specific frequency bands of the HCT voltage signal spectra at both 19 and 82 V_p_ with water in the lumen. The integrated powers are normalized to the mean values at 19 V_p_ within each dataset. Dashed lines represent the median and quantiles. **** indicates statistical significance between groups with *p* < 0.0001 using two-tailed, unpaired *t*-test. Table S1: Material properties of HCT (DL-47) used in simulations.
