# Supplementary material for: Self-Sensing with Hollow Cylindrical Transducers for Histotripsy-Enhanced Aspiration Mechanical Thrombectomy Applications
Source: Sensors (Basel). 2025 Sep 2;25(17):5417. doi: 10.3390/s25175417 (PMC12431187; doi:10.3390/s25175417)
Supplement: Supplementary file 1 [file sensors-25-05417-s001.zip › sensors-3761370-supplementary.pdf]

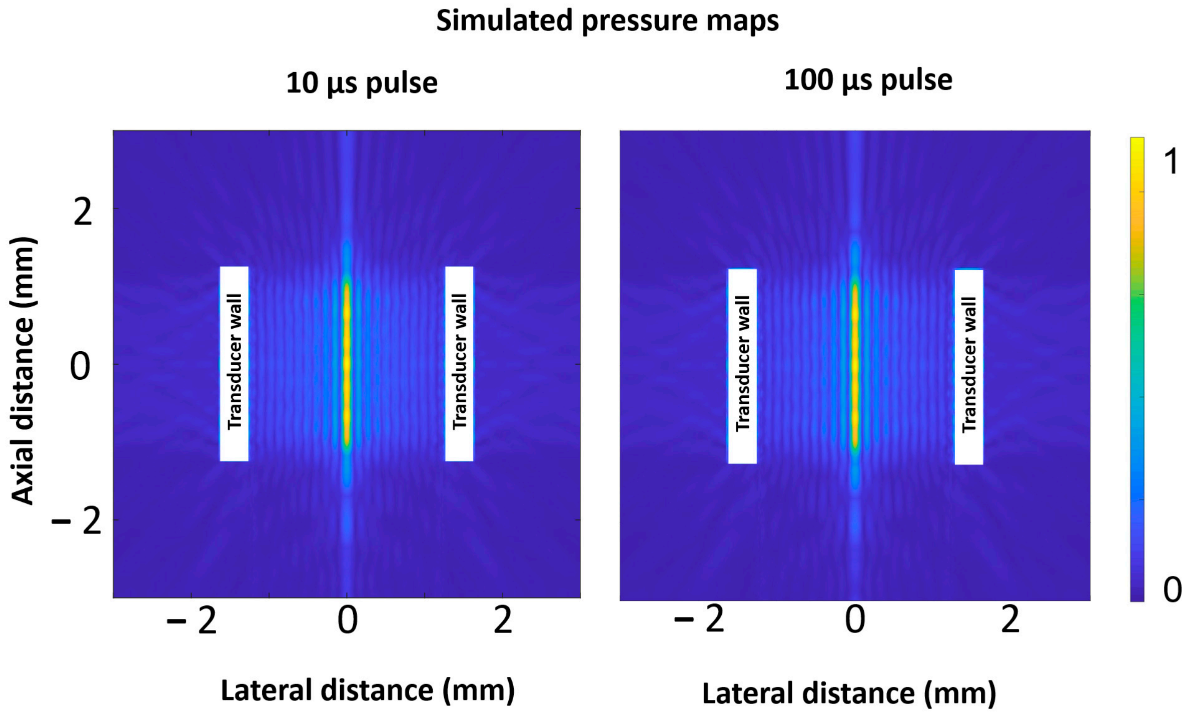

Supplementary Figure S1. Simulated pressure maps using 10 µs (left) and 100 µs (right) pulses at 6.17 MHz in water. Pressures are normalized to the peak pressure within the field.

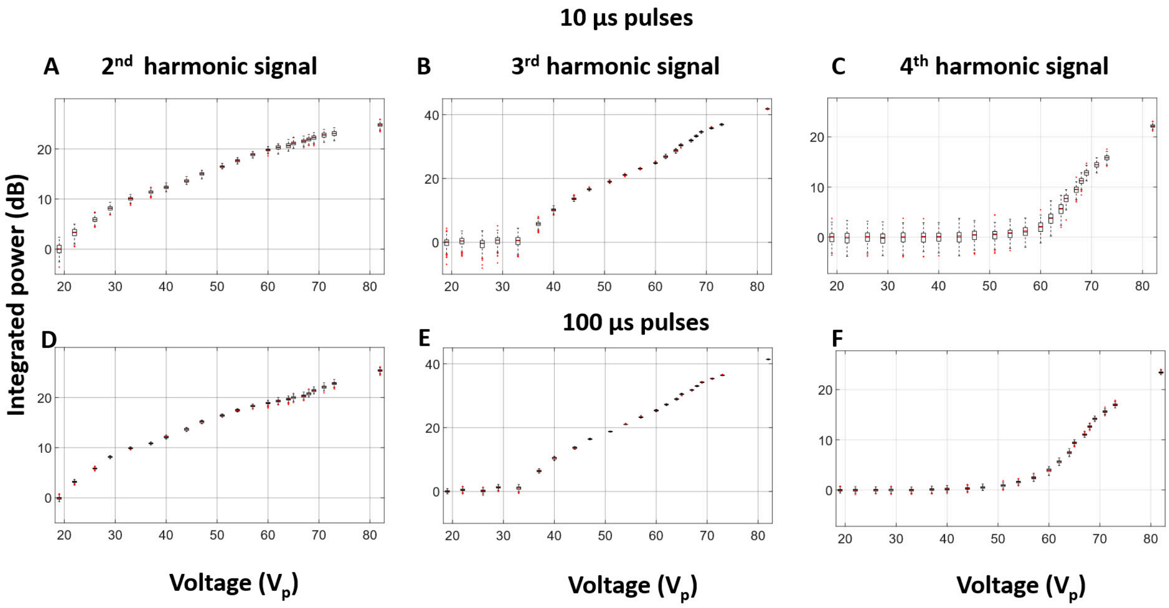

Supplementary Figure S2. Box plots of integrated power of specific frequency bands of the transducer voltage spectra as a function of applied voltage in water for both 10 µs and 100 µs pulses. The 2nd harmonic (A, D) increases in a linear behavior. The 3rd harmonic (B, E) signal exhibits an inflection at a sub-cavitation voltage, while the 4th harmonic (C, F) starts to increase at voltage over the cavitation threshold. The integrated powers are normalized to the mean value at 19 V_P within each dataset.

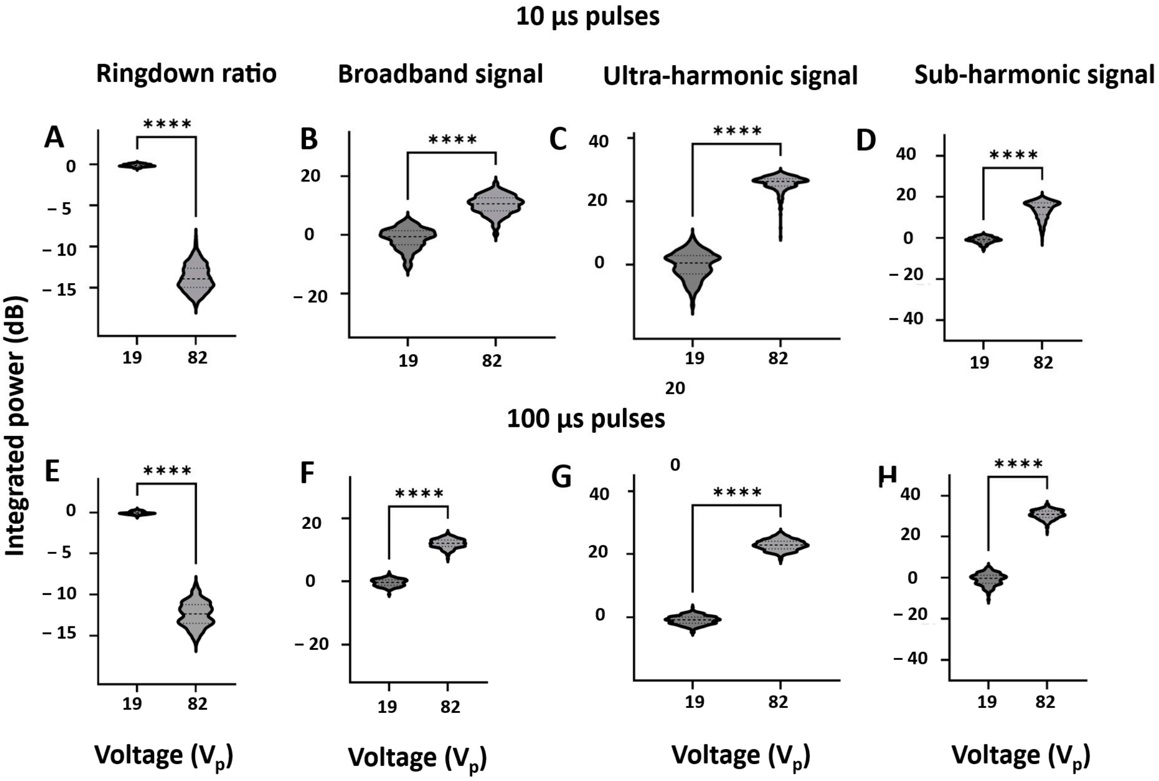

Supplementary Figure S3. Violin plots for both 10 µs and 100 µs pulses comparing the integrated power for (A, E) the ringdown ratio in time domain and the broadband (B, F), ultra-harmonic (C, G), sub-harmonic (D, H) of specific frequency bands of the HCT voltage signal spectra at both 19 and 82 V_P with water in the lumen. The integrated powers are normalized to the mean values at 19 V_P within each dataset. Dashed lines represent the median and quantiles. **** indicates statistical significance between groups with $P<0.0001$ using two-tailed, unpaired t-test.

Supplementary Table S1 Material properties of HCT (DL-47) used in simulations

Mechanical Property	Value	Units
S	7800	kg/m^3
Constant	Stiffness	N/ m^2
C11	1.56e+11	N/ m^2
C12	8.9e+10	N/ m^2
C13	8.8e+10	N/ m^2
C22	1.56e+11	N/ m^2
C23	8.8e+10	N/ m^2
C33	1.32e+11	N/ m^2
C44	3.1e+10	N/ m^2
C55	3.1e+10	N/ m^2
C66	3.4e+10	N/ m^2

Piezoelectric Property	Value	Units
Constant	Stress	
e31	-4.9	c/m^2
e32	-4.9	c/m^2
e33	18.5	c/m^2
e24	14.3	c/m^2
e15	14.3	c/m^2
Polling	X+	

Damping Property	Value	Units
Type	Raleigh	
Damping Units	Q	
Bulk attenuation	650	
Shear attenuation	650	
Loss frequency	1e+06	Hz

Dielectric Property	Value	Units
Constants	Strain	
EpsX	960	
EpsY	960	
EpsZ	870	

Material properties of liner used in simulations

Mechanical Property	Value	Units
Density	1120	kg/m^3
Velocity	2600	ms^{-1}

Material properties of epoxy 301 used in simulations

Mechanical Property	Value	Units
Density	1151.75	kg/m^3
Velocity	2649	ms^{-1}
